# Smoking Behavior and Smoking Cessation Because of and during the COVID-19 Pandemic: A Brief Online Survey 12 Months into the Pandemic and during the Second Wave in Europe

**DOI:** 10.3390/ijerph192416540

**Published:** 2022-12-09

**Authors:** Jule M. Finck, Sabine Bohnet, Katharina Auth, Imke Tangemann-Münstedt, Daniel Drömann, Klaas F. Franzen

**Affiliations:** 1 Medical Clinic III, Campus Lübeck, University Hospital Schleswig-Holstein, 23562 Luebeck, Germany; 2 Airway Research Center North, Member of the German Center for Lung Research (DZL), 22927 Großhansdorf, Germany

**Keywords:** COVID-19, smoking cessation, quit smoking, Italy, Spain, United Kingdom, Germany, second wave, online survey

## Abstract

Smoking is considered a major preventable cause of cardiovascular and lung diseases, as well as cancer. During the COVID-19 pandemic, there was extensive discussion about the influence of nicotine use; ultimately, smoking was considered a major risk factor for poor disease progression. Therefore, in April 2021, we conducted an anonymous cross-sectional online survey on smoking and vaping behavior, as well as smoking cessation, in four different countries in Europe (the United Kingdom, Germany, Spain, and Italy). A total of 3605 participants completed a questionnaire on their smoking and vaping behaviors and smoking cessation because of and during the COVID-19 pandemic. Fear of COVID-19 infection, a high percentage of quarantine stays (44.9% Italy and 52.1% Spain), and high infection (75.5% Italy and 52.4% Spain) and death (42% Italy) rates in respondents’ personal circles were observed mostly in the surveyed populations of Italy and Spain. Smoking cessation attempts and success were mainly seen in the Italian population and were linked to psychological distress, while the same effects were shown for vaping in Spain. In summary, health anxiety was detected in all cohorts. Despite these findings, smoking as a risk factor for severe outcomes of COVID-19 infection did not lead to a higher rate of smoking cessation attempts.

## 1. Introduction

SARS-CoV-2 (severe acute respiratory syndrome coronavirus type 2, responsible for COVID-19 disease) first emerged in Wuhan, China, in December 2019 [1]. Subsequently, the coronavirus spread all over the world, reaching Europe toward the end of December 2019/beginning of January 2020 [2]. COVID-19 was declared a global pandemic by the World Health Organization (WHO) on 11 March 2020 [3]. In this publication, we focus on four countries, the United Kingdom (UK), Germany, Spain, and Italy, which all went through different stages of the pandemic with varying severity levels and timings of the COVID-19 infection waves. There have been several infection waves with different virus variants in these four countries to date. These waves of infections occurred at different times from country to country. In Germany, the periods were as follows: first wave—spring 2020, second wave—autumn/winter 2020/2021, third wave—spring 2021, fourth wave—autumn 2021, and fifth wave—beginning of 2022 [2]. Italy was reporting many new infections and about 1000 deaths a day due to COVID-19 in March 2020 [4]. The restrictions to daily life in the United Kingdom were not that strict in March 2020, resulting in the rapid development of many new infections in the beginning of April [5]. Restrictions such as curfews and mandatory use of face masks followed, especially in Italy and Spain, where people were only allowed to leave their houses for grocery shopping [6]. There were 243,371,671 confirmed COVID-19 infections and 2,057,263 deaths in Europe, and 583,038,110 confirmed COVID-19 infections and 6,416,023 deaths worldwide as of 9 August 2022 [2]. After several mutations and waves of infection, the Omicron subtype currently predominates in Europe. However, the most common symptoms of COVID-19 infection are still cough, fever, and flu-like symptoms. Compared with the survey period, the hospitalization rate is currently significantly lower at the time of writing in relation to both the number of infected people and the mortality caused by COVID-19 [7]. The risk of severe outcomes of COVID-19 infection increases with higher age and/or pre-existing medical conditions such as cardiovascular diseases, COPD (chronic obstructive pulmonary disease), and diabetes mellitus type 2.

Miyara et al. reported in an earlier paper a lower incidence of COVID-19 infections among smokers [8]. Therefore, Jean-Pierre Changeux et al. hypothesized that a current smoking status might be a protective factor against COVID-19 infection owing to the nicotinic acetylcholine receptor. He postulated in April 2020 that this pathomechanism might also be used as a possible therapeutic approach [9]. This thesis that smoking protects against COVID-19 was relevant new information in April 2020. Over the further course of the pandemic, however, new data were condensed and compiled by the Robert Koch Institute, revealing that smoking is a significant risk factor for a severe course of the disease [7].

Smoking continues to be considered one of the most important preventable risk factors for cardiovascular disease, lung disease, and cancer [10]. Because of the behavior’s long-term consequences, European countries are trying to reduce its incidence with the help of various regulatory measures and, above all, prevention programs. The prevalence of smoking differs among the surveyed countries. The United Kingdom has various programs to prevent smoking such as “Smokefree 2030”; as a result of these programs, there are more ex-smokers and fewer active smokers compared with the other surveyed countries (14.4% in 2018) [11]. The remaining three countries have higher smoking rates: 28.3% in Germany, 22.1% in Spain, and 22.4% in Italy (2019) [12]. Particularly in Italy and Spain, smoking is part of the social setting.

Owing to the rapidly changing conditions of the COVID-19 pandemic and their fluctuating incidence, alternative methods for obtaining population responses through surveys are necessary. The data collection phase of population-representative household surveys or telephone surveys takes a long time, which is why these methods do not provide adequate information in these situations [13]. In addition, the response rate of telephone surveys is low [7,14]; therefore, the data must be corrected and adjusted for non-responders [2]. Online questionnaires offer a solution to this problem; in addition to reaching as many participants as possible in a short time, they seem to be particularly suitable for pandemics such as COVID-19 [15].

Therefore, within four European countries with different smoking incidences and pandemic courses, this study aimed to determine the effects of the pandemic on the desire for tobacco cessation, as well as possible causes, such as fear, within the “smoking” group at risk for severe courses of COVID-19. Considering the fast pace of the pandemic, as discussed above, the data collection method was based on an online questionnaire.

## 2. Materials and Methods

### 2.1. Participants

The data collection in this study was based on a cross-sectional online survey conducted in four European countries, which were chosen because of their COVID-19 situations. In addition to it being our own location, we chose Germany primarily because of the measures taken to contain the pandemic, the initially low number of cases, and the average smoking rate compared with the two southern European countries. The United Kingdom was included because of the decreasing rate of cigarette consumption through Smokefree campaigns and the delayed action of the government at the start of the pandemic. Italy and Spain were selected because of the high number of cases and deaths at the beginning of the pandemic, as well as the high number of smokers. Among the four European countries, only the survey from the United Kingdom was based on a sample distributed representatively for age, gender, and ethnicity according to Prolific’s algorithms [16]. The participants were recruited via the research platform Prolific, which is hosted and managed by Prolific Academic Ltd. (Oxford, England). Using a cross-sectional online survey on the Prolific platform, it is possible to collect data from individuals from different countries from all over the world; this study focused on the United Kingdom, Germany, Spain, and Italy. Through Prolific, it was possible to generate a representative sample at a predefined number; thus, a sample size of 1250 was planned to be recruited with an ordinary distribution pattern of sex, age, and ethnicity in the United Kingdom. This preselection on the Prolific platform ensured the existence of a representative cohort. As the preselection sample was only possible for the United Kingdom, a further condition applied for all four countries was that the respondents’ mother tongue or the first language had to match the nationality, e.g., the language “German” was required for the German sample. It was planned to recruit 750 participants for each of the other three countries [17]. The platform Prolific has 123,199 (as of 1 August 2022) active registered individuals, of which 28.79% live in the United Kingdom with the first language “English” (*n* = 35,465). There are fewer people actively registered in Germany (1.54%; *n* = 1909), Spain (1.23%; *n* = 1.512), and Italy (3.35%; *n* = 4.129). Participants receive money as an incentive for completing surveys on the Prolific platform [17]. In our study, the participants could earn GBP 6.12–6.95 (USD 7.5–8.52) per hour, which corresponded to at least GBP 1.00 (USD 1.23) for a completed survey. The average remuneration was at least GBP 11.15 (USD 13.09) per hour per participant.

The study was submitted to the local ethics committee of the University of Lübeck with reference to a previous study using the same methodology (AZ 20-290; date of approval March 2021) [18]. A further assessment was not necessary from the point of view of the ethics committee, owing to the absence of personal data and limited possibility of identification. The study was conducted according to the Declaration of Helsinki, and the Checklist for Reporting Results of Internet E-Surveys (CHERRIES statement) 21 was considered during implementation.

### 2.2. Data Collection

The authors created the questions and decided on the subtopics to be included in the survey. The survey was translated into English, Spanish, and Italian by native speaking medical staff. Subsequently, the questionnaires were retranslated by another person for review. Moreover, each question was checked for practicability and validity. Some questions related to psychological stress during the COVID-19 pandemic were developed or used from surveys from the University of Marburg [19,20]. For reliability testing, a test for internal consistency was carried out with 25 subjects from a university environment, which resulted in a Cronbach’s alpha of 0.86 in the statistical analysis. In the end, the final version of the survey was edited and approved by the local ethics committee. The questionnaire was created on the online portal umfrageonline.de, which served as a tool to create the electronic version of the questionnaire. Then, the questionnaire was uploaded to and linked with the Prolific platform. Adaptation to the platform Prolific was taken into account throughout the development of the questionnaire on umfrageonline.de. Some questions, such as the demographic data, health status, current smoking/vaping status, and COVID-19-related questions (disease and infection, mental health issues during the pandemic, and vaccination), had to be answered by all participants. Detailed questions about smoking/vaping status, including the Fagerström test and smoking/vaping cessation during the pandemic, only had to be answered by active/ex-smokers/vapers and dual users. Most of the questions had to be answered in order to continue the questionnaire, with some exceptions. The period from the beginning of the pandemic to the time of the survey was divided into four sections according to the various government measures taken in Germany (before 27 January 2020, from 27 January to 22 March 2020—first wave, from 22 March to 2 November 2020—second wave, and after 2 November 2020).

The participants only received the reward if they completed the whole questionnaire, including the questions required for their personal status. The German questionnaire was activated from 15 April to 22 April 2021 during the ongoing third wave of the pandemic in Europe, whereas the Italian questionnaire was only open on 15 April, the Spanish one was open from 15 April to 16 April, and the U.K. survey with the representative sample was open from 15 April to 22 April 2021. The British questionnaire was available for at least the first 1250 Prolific users who fit the U.K. representative sample requirements during the recruiting period. Furthermore, Prolific users filtered by “nationality” and “first language” (German, Spanish, and Italian questionnaires) could fill in the questionnaire on a first come, first served basis. A total of 750 participants were planned for each sample. Altogether, 1459 participants were recruited in the U.K. representative sample, along with 726 for the German, 677 for the Spanish, and 706 for the Italian standard samples. In addition to basic demographic and health status data, we asked questions about the participants’ personal history with COVID-19, their opinions and fears, and the status and acceptance of the vaccination against COVID-19. Active/ex-smokers/vapers and dual users were requested to answer questions on their smoking/vaping behavior, their addiction to smoking/vaping with the help of the Fagerström test, and their possible smoking/vaping cessation. Some of the COVID-19 questions, such as past COVID-19 infections, fear, and smoking/vaping cessation attempts, were asked retrospectively.

Furthermore, the practicability and validity were amplified with the help of two attention check questions (i.e., control questions). These two questions had no substantive connection to COVID-19, and they required the participant to fill in a specific and unique answer, which made it clear that the question had been read correctly. With the help of these control questions, random clicking patterns could be reduced and excluded afterward.

### 2.3. Statistical Analysis

**Figure 1:**
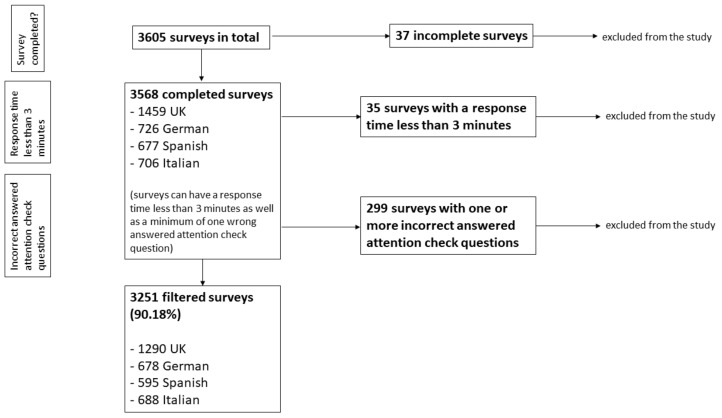
Flowchart of the cleansing process of the survey responses.

To make sure that the data had high credibility and quality, criteria for including or excluding surveys were established. In the first place, only completed questionnaires were accepted for the data analysis; 37 of 3605 surveys had to be excluded because of that. The remaining 3568 participants consisted of 1459 participants from the United Kingdom, along with 726 German, 677 Spanish, and 706 Italian participants. After this first step, a second inclusion criterion was applied, and all surveys with a response time lower than 3 min were excluded from statistical analysis. This inclusion criterion was based on a publication by Geldsetzer et al. [15]. Consequently, another 35 of 3568 participants were excluded. The third stage of data cleansing was related to the attention check questions. Surveys with at least one incorrect answer to the control questions were excluded (299 of the 3568 surveys). Thus, a total of 3251 surveys were filtered and included in the study (1290 British, 678 German, 595 Spanish, and 688 Italian participants), representing 90.18% of the 3605 originally completed surveys (Figure 1).

The data were exported from umfrageonline.de and formatted into a tabulate format in the Microsoft program Excel. The analysis process was conducted with the help of Excel and SPSS (IBM Corp; released 2013; IBM SPSS Statistics for Windows, Version 22.0; Armonk, NY, USA: IBM Corp.). In addition to purely descriptive statistics, we present frequency tables, crosstabs, chi-square tests, *t*-tests, and logistic regression analyses as further statistics. For this publication, we created graphs using GraphPad Prism 5.0 (GraphPad Software Inc., San Diego, CA, USA—United States of America).

## 3. Results

After cleansing, 3251 (90.18%) anonymous surveys were analyzed as a whole group. The data were available for subgroup analysis; therefore, dividing criteria were the home country including first language (the United Kingdom, Germany, Spain, and Italy) and smoking behavior (smoker/vaper, ex-smoker/vaper, never-smoker/vaper, and dual user) of the participants.

### 3.1. General Demographics

**Figure 2:**
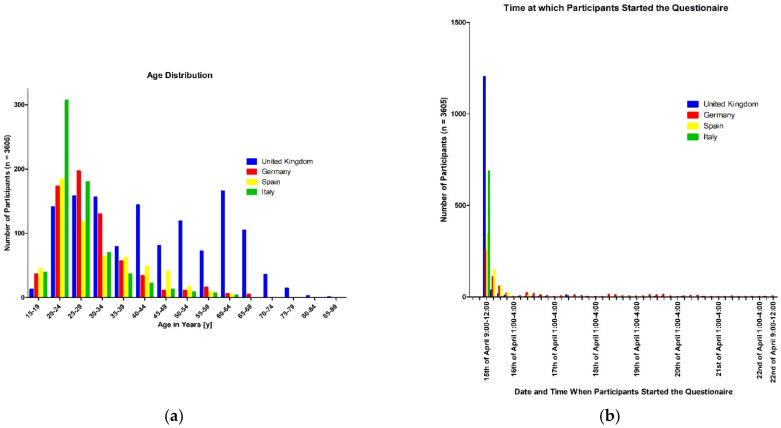
(**a**) Age distribution of participants; (**b**) date and time at which participants started the questionnaire.

In general, the participants who completed the survey between 15 April and 22 April 2021 (Figure 2a) were young, with a mean age of 35 years, while the participants from the United Kingdom stood out, with an older mean age of 44 years (Figure 2b). Slightly more than half of the participants were male (52.2%), whereas 47.1% were female and 0.7% were gender-diverse. Furthermore, 90.4% of all participants classified themselves as belonging to the Caucasian/European ethnic group. However, there were more members of other ethnic groups such as Asian (7.8%), and African (3.1%) in the English-speaking subgroup than in the others, and a high percentage of Hispanics (18.5%) in the Spanish subgroup. Most of the participants (80.5%) had an academic education (Fachhochschulreife (International Baccalaureate) and Abitur (high-school diploma) or bachelor’s or master’s degree). About two-thirds of the whole surveyed group were working in a temporary, half-time, or full-time job, whereas one-quarter of the participants were job-seeking. In total, 40% of the participants earned less than EUR 1500 per month (approximately GBP 1300), while this proportion in the Italian subgroup was 68.3%. Nearly two-thirds of the whole group were unmarried, with higher percentages in Germany (81%) and Italy (84.9%), and at least one-quarter of all participants were married, with an especially high percentage in the United Kingdom (43.3%). In total, 28.1% of the participants had children; this differed widely among the different countries, where 49.3% of respondents in the United Kingdom had children compared with only 7% in Italy.

### 3.2. General State of Health and COVID-19-Related Medical History

In this population, 21.3% of the participants suffered from mental illnesses and/or psychological stress, followed by hormonal and/or metabolic diseases at 10.3%. In terms of the cross-country comparison, Germany had the highest percentages of these diseases (29.6%) according to the frequency table. Regarding mental illness and/or psychological stress, Italy took second place (25.4%) in the frequency table.

An average of 8.8% of the people surveyed in April 2021 had previously contracted a COVID-19 infection (*n* = 285) (Table 1).

Germany had the lowest infection rate with 4.4% at this timepoint. In the smoker subgroup, the low infection rate of 6.6% was striking, whereas the vaper subgroup and the dual user subgroup showed higher infection rates of 12.4% (dual users) and 16.9% (vapers). These higher infection rates in both dual users and vapers were significantly different according to the cross-table in the chi-square test (*p* < 0.05).

Tiredness, headache, cough, fever, and loss of smell and taste were most frequently stated as the main COVID-19 symptoms (frequency table), and only 4.9% of the sick participants (285) had to be treated in a hospital because of their infection. In particular, in Spain (52.1%) and Italy (44.9%), a large proportion of the surveyed population had been required to quarantine at least once during the pandemic, while the United Kingdom and Germany were a lot less affected. Only 11.4% of all the participants regarded themselves as members of a group officially identified as having an increased risk of severe illness from COVID-19; the percentage was 17.8% in the United Kingdom, which might be related to the higher mean age of the representative group and was significantly different according to the cross-table in the chi-square test (*p* < 0.05). There were more COVID-19 infections in the personal circles of the Italian participants (75.5%; *p* < 0.05) and more of these people had a severe progression resulting in hospitalization (28.7%; *p* < 0.05) or death (42%; *p* < 0.05).

**Figure 3:**
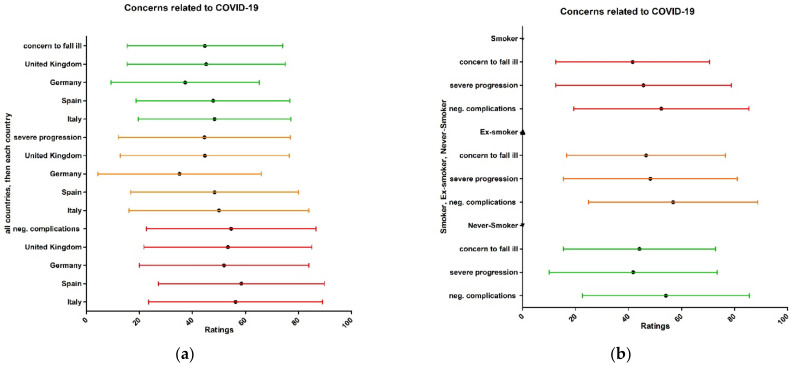
(**a**) Health concerns about falling ill with COVID-19 and nationality subgroup analysis; (**b**) health concerns about falling ill with COVID-19 and nationality subgroup analysis (ranging from 0 for completely unconcerned to 100 for very worried).

The average concern with respect to falling ill with COVID-19 (44.7 from 100), experiencing a severe progression of COVID-19 (44.5 from 100), and having long-term negative complications for physical health (54.6) received more points (concern was greater) from the English-speaking, Spanish, and Italian surveyed populations than from the German population (37.3 from 100, 35.2 from 100, and 51.9 from 100) (Figure 3a). For the subgroup analysis for smokers, ex-smokers, and those who had never smoked, there was no statistical significance; thus, we only give the results of the frequency table in Figure 3b.

In the retrospective survey for the period 2 to 4 weeks before the questionnaire, the results for Spain and Italy showed that there was psychological stress. This psychological stress expressed itself through nervousness, anxiety, or tension, anxiety about the future, and not being able to enjoy life. In the questionnaire, a maximum of 28 points was possible (most affected by medical conditions); Italy and Spain scored 15.7 and 14.8 points, respectively, whereas the United Kingdom and Germany scored fewer points. The chi-square test showed significant differences between countries (*p* < 0.05). Negative correlations of fear versus age (*p* < 0.01) and fear versus income (*p* = 0.019) were seen, suggesting that older people tended to have less fear during the pandemic, as did people with higher incomes. Women tended to be more afraid, although this did not reach significance.

Furthermore, 26.3% of all participants were vaccinated at the time the survey took place; it is worth noting that the participants from the United Kingdom already had a vaccination rate of 51.7% owing to the supply of the AstraZeneca vaccine, which led to a significant difference from the other countries (*p* < 0.05). Moreover, 86.5% of the participants would be vaccinated if given the chance, 9.2% were unsure, and 4.3% would refuse the vaccine. The many infections in the personal circle of Italians were the main reason for Italian participants being vaccinated. For Italy and the United Kingdom, the number of infected people and the number of people who had died from COVID-19 in respondents’ personal circles had a great impact on their decision to be vaccinated, as shown in the linear regression. In general, the number of illnesses/deaths played a significant role in respondents’ decision to be vaccinated (*p* < 0.001). Additionally, 94% of those with a larger number of illnesses/deaths in their personal circle reported the desire to be vaccinated against COVID-19 (*p* < 0.001). No statistical correlation could be seen between being infected by COVID-19 and willingness to be vaccinated. Of the group of participants who were already ill, a disproportionate share showed a trend of not being vaccinated without reaching a significant difference (*p* = 0.058). Ex-smokers/vapers were vaccinated more often than average (*p* < 0.001), whereas nonsmokers rejected vaccination against COVID-19 less often (*p* < 0.001). Above-average numbers of active smokers/vapers would refuse vaccination (*p* < 0.05). A very clear correlation between risk group and willingness to be vaccinated (*p* < 0.001), along with a lower rejection rate of vaccination (*p* < 0.05), was observable.

### 3.3. Subgroup Analysis of Smoking/Vaping Behavior and Cessation

Data were further analyzed by dividing the participants into subgroups according to their smoking/vaping behaviors. The percentage of smokers was higher in the Spanish (29.1%) and Italian (25%) surveyed populations than in the United Kingdom (13.4%) and German (17.6%) populations (*p* < 0.05 according to chi-square test). In the smoking subgroup, 8.49 cigarettes per day and 14.15 pack years were the average, while the smokers in the United Kingdom had higher numbers in terms of cigarettes per day (11.3; *p* < 0.05) and pack years (23.25; *p* < 0.05) compared with other countries. The mean percentage of vapers in the surveyed population was 10.2%, according to the frequency table. Furthermore, 90.6% of the vaping subgroup used e-cigarettes, while the other 9.4% used e-shisha. The mean number of vaping years was 3.1. Moreover, 67.5% of vapers used fluid that contained nicotine, and the amount of fluid used varied as follows: <5 mL (40%), 5–10 mL (43.1%), and 10–15 mL (13.1%). The Spanish participants had the highest percentage of dual users in their group (20%) compared with the mean of 7.9% (*p* < 0.05). The dual user subgroup had a similar number of smoking/vaping years, number of cigarettes per day, and percentage of usage of fluid containing nicotine to the smoker and vaper subgroups. The amount of fluid used was slightly less. The strength of addiction to smoking was measured with the help of the Fagerström test and by an adaption of the Fagerström test for vaping. The vaping subgroup showed a stronger addiction to vaping than the smoking subgroup to smoking. The dual user subgroup had the strongest addiction to smoking but a weaker addiction to vaping. In general, the rate of addiction was rather low, with at least two-thirds of the smokers/vapers/dual users having points ranging from 0 to 4 (low and medium addiction).

Prior to 27 January 2020 (first COVID-19 case in Germany), 31.5% of the smoker subgroup had made one or more smoking cessation attempts, and this proportion even reached 40.8% among Italian smokers. The highest success rates in smoking cessation were seen in Italy (15.4%) and the United Kingdom (14%), which were significant according to the chi-square test. Italy also showed the highest percentage of smoking cessation attempts and their success in the following time periods (*p* < 0.05): from 27 January to 22 March 2020 (first lockdown in Germany), from 22 March to 2 November 2020 (second lockdown in Germany), and after 2 November 2020. The attempt and success percentages decreased during these time periods, with a mean smoking cessation attempt percentage of 9.7% and a mean success rate of 2.8% after 2 November 2020 (Table 2). In the vaping subgroup, the vaping cessation attempts and their success were mainly dominated by Spanish vapers (according to the cross-table and the chi-square test, *p* < 0.05), as well as by Italian vapers in some time periods. In the dual user subgroup, the country with the most smoking and vaping cessation attempts varied across different time periods, but the success in quitting smoking/vaping, according to self-disclosure, remained strongest in Germany and weakest in Italy without reaching significance after adjusting for the days (*p* > 0.05) while comparing the different time periods. In total, the desire for tobacco cessation was significantly higher in the Italian and Spanish groups (*p* < 0.01). This higher desire for tobacco cessation was also significantly associated with the level of academic education (higher education, *p* = 0.012), employment status (full-time occupation, *p* < 0.001), and marital status (married, *p* < 0.001), and it showed a trend without reaching significance depending on the net income (higher income, *p* = 0.05). Weak effects on a wish to quit tobacco usage could be seen in groups of participants with greater concern about falling ill with COVID-19 (*p* = 0.002), experiencing a severe case of COVID-19 (*p* = 0.004), and having long-term negative complications from COVID-19 (*p* = 0.001). Taking all of this into account, we created a logistic regression model considering age, gender, income, and infection with COVID-19 as the variables. The highest significance could be seen in the factor of age, with younger participants tending to have a stronger wish to quit smoking tobacco.

## 4. Discussion

### 4.1. Discussion

In the period after the second wave of infections, this study investigated whether smokers and vapers in four European countries (the United Kingdom, Germany, Spain, and Italy) were concerned about the risk of a more severe course of infection and were more likely to quit smoking.

Compared with the general public, the study populations in Germany, Spain, and Italy had a lower mean age. The mean age for the United Kingdom (44.1 years) was slightly above the U.K. average, which can be attributed to the representative sample [21,22]. In addition to age, the level of education also differed from the normal population, with a larger number of academics [23]. On the other hand, there was an increased number of unemployed participants in the population cohort surveyed compared with the normal population [24,25]. The mean income of the study population was lower than the mean income of the general public of the four analyzed countries, with the most striking disparity seen in Italy, where 40.3% of participants had an income of less than EUR 500 a month [26,27]. This observation can be explained by the use of this platform to earn money without much effort at a low threshold or on the side as a side job. Another reason could be the young mean age of the study population and that these participants had yet to complete their studies and, therefore, had only low-income jobs. The age distribution also fits in with the preparatory work from our own working group [18], explaining the affinity for technology in this cohort.

Every fourth person in Europe suffers from mental illness, which can also be seen in our study population (21.3% of participants); however, the percentages in different countries in Europe vary [28].

The percentage of study participants who had been previously infected with SARS-CoV-2 was 8.8%, which was lower than the average of approximately 11.32% of the European population who had been infected at the time of the survey (WHO data of 19 April 2021) [2,29]. In the German surveyed population, the percentage of COVID-19 infections was higher (4.4%) than in the general German population (around 3.67%) [2,30]. In general, the infection rate in the surveyed German population was low (4.4%) in comparison with the three other analyzed countries (around 10%). The low infection rate of the smoker subgroup (6.6%) brings up the question of whether smoking, particularly nicotine, is a protective factor against COVID-19 infection, which was previously discussed in a French study by Jean-Pierre Changeux et al. In this study, the authors set up the hypothesis that the nicotinic receptor, which is sensitive to nicotine and acetylcholine, might also be a target for COVID-19 infection and that nicotine might, therefore, be a protective factor against COVID-19 infection because it uses the same target receptor [9]. However, the infection rates in the vaper subgroup (12.4%) and dual user subgroup (16.9%) were higher than in the general mean European population. More than two-thirds of the vaper subgroup used fluid containing nicotine, which could challenge the theory of nicotine being a protective factor against COVID-19 infection. Other reasons for the high infection rates in these subgroups are possible. Owing to the young age and the low percentage of cardiovascular and lung diseases in the surveyed population, only 4.9% of the sick patients (285 COVID-19 infections in total) had to be treated in hospital [7].

Various characteristics could explain the greater concern within the surveyed cohort, especially in the Italian and Spanish populations. These include the ordered quarantine (Spain 52.1% and Italy 44.9%), the large number of COVID-19 infections (Spain 52.4% and Italy 75.5%), and the hospital treatments and deaths within respondents’ circles of relatives and acquaintances. The lower fear in older participants and/or in participants with higher incomes may be a consequence of their longer experience in dealing with problems and their safe financial status in times of crisis. Young people, especially women, tend to have more anxiety in times of crisis, which could be related to the fear of losing older relatives without being able to help. In addition, media coverage and the death toll at the beginning of the pandemic also played a decisive role, especially in the most affected countries [6,31,32]. The government of the United Kingdom reacted more slowly than the Italian and Spanish governments in terms of setting restrictions in daily life, which led to a substantial development of COVID-19 infections and deaths in the beginning of April 2020 [2,33]. The different extents of the measures applied to contain the pandemic and the corresponding smaller number of deaths also ensured that the fear of illness or a severe course was significantly lower in the German cohort compared with the other subgroups [2]. In addition, the availability of hospital beds and the passable supply of functioning medical equipment played a decisive role. Only a small percentage of all participants (11.4%), including Italy and Spain, saw themselves as part of a group officially identified as having an increased risk of severe illness from COVID-19, even though their general fear of COVID-19 was not low.

However, the Italian and Spanish participants were particularly affected by medical conditions such as nervousness, anxiety or tension, anxiety about the future, and not being able to enjoy life 2–4 weeks before the survey started. This was shown through a test in which the participants had to state the frequency of seven symptoms experienced in the last 2–4 weeks before the survey, with a total maximum of 28 points possible. Italy and Spain scored the most points in this test, whereas the United Kingdom and Germany scored lower. An Italian study from Lasalvia et al. [34] and a Spanish study from María Dolores Ruiz-Fernández et al. [35] demonstrated a growing psychological impact of the pandemic on the psychological health of healthcare workers within their countries, leading to increased psychological distress. In general, the number of thoughts about the pandemic changed with the activity of the pandemic, whereby respondents’ thoughts about COVID-19 in summer 2020 were less frequent than at the time of the present survey in April 2021, which marked the third wave of the pandemic, except in the United Kingdom. This concern and psychological distress were also understandably expressed in the rates of vaccination, wherein the possibility of supply also played a decisive role, as was shown for the United Kingdom and the Astra-Zeneca vaccine. Psychological distress, belonging to the group at risk of a severe course of COVID-19, and smoking status (ex-smoker/vaper and nonsmoker) were associated with a stronger wish to be vaccinated, which could be explained by a higher health awareness.

The surveyed populations of Spain and Italy smoked more than the general populations in these countries: 29.1% versus 22.1% for Spain and 25% versus 22.4% for Italy [12]. While analyzing the smoking/vaping behaviors of the different subgroups, the low number/volume of cigarettes/fluid and the low number of smoking/vaping years were striking. Moreover, the addiction to smoking/vaping was rather weak in the subgroups, with at least two-thirds of the smokers/vapers/dual users scoring 0–4 (low or medium addiction) in the Fagerström test.

Despite, or precisely because of, this low or moderate dependence, certain changes in tobacco cessation were recorded for individual cohorts. The Italian participants in the smoking subgroup showed the highest percentages of smoking cessation attempts and self-reported success. High percentages of vaping cessation attempts and their self-reported success were mainly reported by Spanish vapers, as well as by Italian vapers in some time periods. The dual user group had varying levels of smoking/vaping cessation attempts, but the German dual users had the most self-reported success. The attempt and success percentages were reduced during these time periods, taking into account their variable length. Consequently, fewer participants (lower percentage) attempted to quit smoking in a shorter period of time. The reported success in connection with smoking cessation attempts can be explained, among other things, by the possible influence of COVID-19 and by the academic level of the survey population. It is also important to note that the data on smoking cessation attempts were based only on participants’ recollections, which may have been inaccurate.

One the one hand, one would expect the success rates to increase because the people were afraid of COVID-19 infection, whereas, on the other hand, people use smoking/vaping to calm down in stressful situations [36], as well as for socializing during events, which were restricted during the pandemic. In the surveyed population, the people with the greatest fears with respect to COVID-19 were the Italian and Spanish participants, which might be related to the fact that the Italian participants made the most smoking cessation attempts and the Spanish participants made the most vaping cessation attempts. In the United Kingdom, the success of smoking cessation attempts was mostly a consequence of nationwide campaigns such as the “Smokefree 2030” program, which were established and started before the pandemic, rather than a consequence of the fear of COVID-19 [11]. Accordingly, during the pandemic, the rates of smoking cessation attempts were lower. Furthermore, the slow action of the U.K. government at the beginning of the pandemic might have been a reason for the population in the United Kingdom not taking COVID-19 too seriously and not seeing a point in acting on their own. In Germany, the fear of COVID-19 was generally lower than in the other three countries, which might be a result of the lack of pressure to change lifestyle habits among the German participants. With higher levels of academic education, full-time employment status, and marriage, the desire for tobacco cessation increased, which might be related to smoking/vaping being less tolerated in social settings and the knowledge about the health risks of smoking/vaping in general. In addition, target-oriented research on COVID-19 combined with smoking/vaping health risks and participants informing themselves and their families could have led to the desire for tobacco cessation during the pandemic. This same wish was predominantly stronger in younger groups, which may be a result of the growing health awareness owing to social media and the wish to live a long life without health issues caused by smoking/vaping.

### 4.2. Limitations

This study has several limitations. First, the survey was representative for the United Kingdom only, and not for Germany, Spain, and Italy in terms of the age, educational level, income level, and smoking/vaping status of the participants. Most participants were younger than 30 years. All cohorts had a higher educational level than the general population. Additionally, the participants were recruited online with the help of a survey platform, making it easier for younger, more technologically competent participants to participate. Smoking/vaping years were low owing to a younger mean age of the participants and the addiction to smoking/vaping in the smoker/vaper/dual user subgroups (Fagerström test), which may have been a reason for the greater desire for smoking cessation. Another limitation of the survey was the self-assessment of smoking cessation and the risk for a severe course of infection. Therefore, there was a risk of memory bias (e.g., recency effect). Unfortunately, these results could not be mapped via POCT tests or CO measurements. In addition, we had no information on past medical history to validate the plausibility of the participants’ assessments. Despite the attention check question, the question of the validity and plausibility of the data in general remains. The financial incentive of the questionnaire could have led to participants not thinking long enough about their answers.

## 5. Conclusions

In summary, this survey provided information on the general smoking/vaping behaviors and how the sentiments and fears toward COVID-19 changed these behaviors through smoking/vaping cessation attempts in the U.K., German, Spanish, and Italian populations. All cohorts were more educated and technologically competent than the public average, as well as younger than the general population, with the exception of the United Kingdom. It was confirmed that online surveys can collect information about rapidly changing situations in a short time in different European countries, which makes the comparison more valid [12]. This study pointed out that people need more support and help in crisis situations such as the COVID-19 pandemic. Leaving people alone with their concerns during quarantine with their fear of losing loved ones, as well as seeing dramatic pictures on TV while having fewer people to talk to about it, stimulates psychological distress and makes people more prone to mental illnesses in the future. Of course, the observed increase in smoking/vaping cessation attempts with success among participants in Italy and Spain at the beginning of the pandemic compared with other countries can be positively assessed, but it may not have been the consequence of psychological distress; additional factors include government-established campaigns such as “Smokefree 2030” in the United Kingdom. In stress-free situations, it is easier to stop smoking/vaping because the calming effect of smoking/vaping is less needed, as was visible in the overall decreasing number of attempts and their success during the COVID-19 pandemic. In general, more education and targeted promotion to increase the acceptance of vaccination among nonvaccinated groups needs to be established, and further research has to be conducted to understand why smokers refuse vaccination. After 2 years of living with the threat of COVID-19 and less media presence of the disease, it might now be interesting to see how smoking/vaping behaviors have developed, while also considering the impact in Europe of inflation and, consequently, higher living expenses.

## Figures and Tables

**Table 1:** COVID-19-related medical history (*N* = 3251 after excluding ineligible participants).

Medical History	All Countries (*n*, %)	United Kingdom (*n*, %)	Germany (*n*, %)	Spain (*n*, %)	Italy (*n*, %)
SARS-CoV-2 infection	285 (8.8%)	130 (10.1%)	30 (4.4%)	60 (10.1%)	65 (9.5%)
Hospitalization	14 (0.8%)	1 (0.1%)	1 (0.4%)	9 (2.6%)	3 (0.7%)
Quarantined	1128 (34.8%)	345 (26.8%)	165 (24.4%)	310 (52.1%)	308 (44.9%)
Risk group (self-assessed)	371 (11.4%)	230 (17.8%)	74 (10.9%)	45 (7.6%)	22 (3.2%)

**Table 2:** Smoking cessation attempts within the smoker subgroup, *n* = 466.

Smoking Cessation (Attempts and Success)	All Countries *n* = 466 (%)	United Kingdom *n* = 114 (%)	Germany *n* = 104 (%)	Spain *n* = 118 (%)	Italy *n* = 130 (%)
Prior to 27 January					
Attempts	31.5%	29.8%	20.2%	33.1%	40.8%
Success	11.8%	14.0%	9.6%	7.6%	15.4%
27 January–22 March					
Attempts	11.6%	4.4%	3.8%	12.7%	23.1%
Success	5.4%	2.6%	1.9%	5.1%	10.8%
22 March–2 November					
Attempts	12.7%	6.1%	9.6%	16.1%	17.7%
Success	5.4%	3.5%	2.9%	5.1%	9.2%
After 2 November					
Attempts	9.7%	7.0%	6.7%	9.3%	14.6%
Success	2.8%	2.6%	1.0%	2.5%	4.6%

## Data Availability

All relevant data are presented in this paper, and the raw data presented in this study are available upon request from the corresponding author.
